# Genome-wide transcription analysis of histidine-related cataract in Atlantic salmon (*Salmo salar* L)

**Published:** 2009-07-09

**Authors:** Christiane Tröße, Rune Waagbø, Olav Breck, Anne-Kristin Stavrum, Kjell Petersen, Pål A. Olsvik

**Affiliations:** 1 National Institute of Nutrition and Seafood Research (NIFES), Bergen, Norway; 2 Marine Harvest Norway AS, Bergen, Norway; 3 Department of Clinical Medicine, University of Bergen, Bergen, Norway; 4 Computational Biology Unit (CBU), Bergen Center for Computational Science (BCCS), University of Bergen, Bergen, Norway

## Abstract

**Purpose:**

Elevated levels of dietary histidine have previously been shown to prevent or mitigate cataract formation in farmed Atlantic salmon (*Salmo salar* L). The aim of this study was to shed light on the mechanisms by which histidine acts. Applying microarray analysis to the lens transcriptome, we screened for differentially expressed genes in search for a model explaining cataract development in Atlantic salmon and possible markers for early cataract diagnosis.

**Methods:**

Adult Atlantic salmon (1.7 kg) were fed three standard commercial salmon diets only differing in the histidine content (9, 13, and 17 g histidine/kg diet) for four months. Individual cataract scores for both eyes were assessed by slit-lamp biomicroscopy. Lens N-acetyl histidine contents were measured by high performance liquid chromatography (HPLC). Total RNA extracted from whole lenses was analyzed using the GRASP 16K salmonid microarray. The microarray data were analyzed using J-Express Pro 2.7 and validated by quantitative real-time polymerase chain reaction (qRT–PCR).

**Results:**

Fish developed cataracts with different severity in response to dietary histidine levels. Lens N-acetyl histidine contents reflected the dietary histidine levels and were negatively correlated to cataract scores. Significance analysis of microarrays (SAM) revealed 248 significantly up-regulated transcripts and 266 significantly down-regulated transcripts in fish that were fed a low level of histidine compared to fish fed a higher histidine level. Among the differentially expressed transcripts were metallothionein A and B as well as transcripts involved in lipid metabolism, carbohydrate metabolism, regulation of ion homeostasis, and protein degradation. Hierarchical clustering and correspondence analysis plot confirmed differences in gene expression between the feeding groups. The differentially expressed genes could be categorized as “early” and “late” responsive according to their expression pattern relative to progression in cataract formation.

**Conclusions:**

Dietary histidine regimes affected cataract formation and lens gene expression in adult Atlantic salmon. Regulated transcripts selected from the results of this genome-wide transcription analysis might be used as possible biological markers for cataract development in Atlantic salmon.

## Introduction

A cataract is defined as the loss of transparency of the eye lens. The eye lens is composed of two types of cells, an outer monolayer of epithelial cells and underlying fiber cells, which are nourished by the outer monolayer. As the lens grows, epithelial cells differentiate into fiber cells covering the older layers of fiber cells like the skins of an onion. The fiber cells eventually lose their nuclei and other organelles. The further the fiber cells are from the epithelial cells, the lower the metabolic activity. The fiber cells contain the major lens proteins, the crystallins. These proteins are highly ordered and tightly packed, which enables light to pass through the clear lens and to be absorbed by the retina where vision occurs [1]. Cataracts can be caused by a variety of factors including physical damage, oxidative stress, age, and genetic predisposition. Several nutrient deficiencies have been found to provoke cataracts. Since cataracts are a major problem for humans, especially elderly people, several mammalian models, mostly rodents, have been developed to study the disease. However, cataracts are not unique to mammals. They have also been observed in populations of wild and farmed fish, mainly Atlantic salmon (*Salmo salar* L) [2]. For the fish farming industry, this constitutes a serious problem with the potential for economic losses. Affected fish have reduced growth rates and increased susceptibility to secondary diseases compared to healthy fish [3]. Numerous nutritional factors have been related to cataract formation in farmed fish [4], and during the last few years, advances in feed composition have reduced both the incidence and severity of cataract outbreaks.

Dietary levels of the essential amino acid histidine (His) above the suggested minimum requirement for salmonids of 7 g His/kg diet [5] have been found to prevent or slow the progression of cataract development in Atlantic salmon smolts [6-9]. The His derivative N- acetyl histidine (NAH) is a major component of the salmon lens free amino acid pool. Lens NAH concentrations directly reflect dietary His levels, and NAH has therefore been established as a lens-specific marker for the His status of salmon [6,9]. It has been proposed that NAH may act as an osmolyte in the goldfish lens, transporting water out of the cell along the NAH gradient followed by immediate hydrolysis and active uptake of acetate and His back into the cell [10]. Studies with Atlantic salmon have supported a role of NAH in lens water homeostasis, although the exact mechanism remains unknown [6]. Additional possible cataract preventative functions of His and His-related compounds include anti-oxidation [11,12], anti-inflammation [13], anti-glycation [14], and buffering capacity [15].

However, at present, it is still unclear how His prevents or mitigates cataract development in salmon, and the molecular basis of cataractogenesis in the salmon lens is unclear. Increased knowledge of these underlying mechanisms would enable us to better advise the fish farming industry on how to eliminate risk factors leading to cataract development, especially in connection with the increased inclusion of alternative feed resources in aquaculture. This would not only improve fish welfare but may also increase fish production with low additional cost. Research performed in teleost fish may also contribute to our understanding of cataract development in higher vertebrates including humans.

The aim of this study was to shed light on the mechanisms by which dietary His prevents or delays cataract development in Atlantic salmon. Using microarray analysis of the transcriptome in lenses of salmon that were fed diets with different His content, we screened for differentially expressed genes in search for a model explaining cataract development in salmon and possible markers for early cataract diagnosis.

## Methods

### Fish feeding experiment

The feeding experiment was performed at Lerang Research Station (Lerang, Norway). The experimental procedures were approved by and animals handled according to the guidelines of the Norwegian State Commission for Laboratory Animals. Atlantic salmon in their second year in sea with a mean start weight of 1,662 g (n=1,834) were fed three diets containing low (L), medium (M), or high (H) levels of His (L: 9 g/kg diet; M: 13 g/kg diet; H: 17 g/kg diet) in duplicate sea net pens. The diets were based on a commercial feed and had a similar overall composition (protein: 375 g/kg; fat: 342 g/kg; ash: 73 g/kg; moisture 83 g/kg). The trial, which was run from June to October, 2006, was divided into three experimental periods defined by two intermediate sampling points in July and September in addition to start and end point sampling. At all sampling points, tissue was sampled and cataract status diagnosed by slit-lamp biomicroscopic inspection of both eyes. The cataract score per lens was assessed on a scale from zero (clear lens) to four (completely clouded lens), summing up to a possible maximum score of eight per fish [16]. We screened for differences in the lens transcriptome in two selected dietary groups, the low-His group LLL (diet L during all three experimental periods; sampled after the third period) and the medium-His group MMM (diet M during all three experimental periods; sampled after the third period). Each dietary group contained 11 biological replicates.

### Tissue sampling

The fish were anesthetized with metacaine and killed by a blow to the head. The lens was dissected quickly after opening the cornea by an incision along the limbus. Muscle tissue attached to the lens was removed, and the lens was cleaned of aqueous humor by rolling it gently on bench paper. The lens was then immediately frozen in a 2 ml RNase-free microcentrifuge tube by placing the tube on dry ice. Of each sampled fish, the right eye lens was used for RNA extraction while the left eye lens was used for NAH analysis. The lenses were stored at -80 °C until RNA isolation.

### NAH analysis

Lens NAH concentrations were analyzed by isocratic reverse phase high performance liquid chromatography (HPLC) with ultraviolet (UV) absorbance at 210 nm using external standard calibration as previously described by Breck and coworkers [9].

### RNA purification

The samples were homogenized on day one using a Retsch MM 301 homogenizer (Retsch Gmbh, Haan, Germany) and were then further processed on the four successive days in randomized order. The number of samples belonging to each group was balanced for each of the four days. Total RNA was extracted using TRIzol reagent (Invitrogen, Carlsbad, CA). Genomic DNA was eliminated from the samples by DNase treatment (DNA-free; Ambion, Austin, TX). RNA for microarray analysis was further purified using the RNeasy MinElute Cleanup Kit (Qiagen, Hilden, Germany). The amount and purity of the isolated RNA was measured with a NanoDrop ND-1000 UV-Vis Spectrophotometer (NanoDrop Technologies, Wilmington, DE). The A260/A280 ratios lay between 2.08 and 2.12 for all RNA samples. RNA quality was determined with the Agilent 2100 Bioanalyzer (Agilent Technologies, Palo Alto, CA). One of the samples had a RNA integrity number (RIN) of 7.9, and the others lay between 8.1 and 9.2. The isolated RNA was stored at -80 °C.

### Microarray experiment

A common reference design with a pool of all RNA samples as the reference was used for the two-channel microarray experiment. All samples were labeled with Cy5, and the reference was labeled with Cy3. The RNA was hybridized to 16K GRASP v. 2.0 arrays [17] on a Tecan HS 4800™ hybridization station (Tecan Group Ltd., Männedorf, Switzerland). The arrays were scanned with a Tecan LS Reloaded scanner (Tecan Group Ltd.) and analyzed using the Axon GenePix 5.1 software (MDS Inc., Toronto, Canada).

The raw data were filtered and normalized using J-Express Pro v.2.7 [18]. The foreground signal intensity values for each channel were extracted per spot from the data files, and all empty, flagged, and control spots were filtered out before the data were normalized using a nonlinear normalization method, global lowess [19]. After normalization, weak spots with a foreground signal intensity below the sum of the background signal intensity and 1.5 times the standard deviation of the background signal intensity in at least one channel were filtered out. All arrays were compiled into a single expression profile data matrix (gene by sample) containing the log ratio of the two foreground signal intensities. Eexpressed sequence tag (EST) clones with more than 30% missing values were removed from the analysis. Missing values were estimated and replaced using the method introduced by Bø et al. [20], LSimpute_adaptive.

Correspondence analysis (CA) [21], significance analysis of microarrays (SAM) [22], and hierarchical clustering of samples and transcripts were performed on the sub-data sets in J-Express. Functional annotation of the transcripts in the data sets was done using the Blast2GO platform [23]. The Gossip tool [24] integrated in Blast2GO was used for functional enrichment analysis applying Fisher's exact test. The microarray experiment was designed to comply with the Minimum Information about a Microarray Experiment (MIAME) guidelines [25]. The applied protocols and final results were uploaded to BASE. MIAME-compliant microarray data were finally uploaded to the ArrayExpress database (accession number: E-TABM-678).

### Quantitative real-time PCR

The results of the microarray experiment were validated by two-step quantitative real-time polymerase chain reaction (qRT–PCR) of selected transcripts that were up-regulated or down-regulated in the low-His group. Primers were designed within the coding sequences using Primer3Plus [26]. Isoform-specific primers were used to amplify sodium/potassium-transporting ATPase subunit alpha-1C (*ATPA1C*) [27]. We tested four potential reference genes that had shown constant expression rates among the experimental groups in the microarray experiment. Three of them have been previously used as reference genes in qRT–PCR analysis in Atlantic salmon [28]. An overview over the target genes and the respective PCR primers is given in Table 1.

**Table 1:** Transcript names, short names, accession numbers, primer sequences, amplicon sizes, and fold change expression values obtained by microarray and qRT–PCR for selected transcripts.

**Transcript name**	**Short name**	**Accession Number**	**Forward primer**	**Reverse primer**	**Amplicon size**	**FC by microarray**	**FC by qRT–PCR**
40S ribosomal protein S20	*RPS20*	BG936672	GCAGACCTTATCCGTGGAGCTA	TGGTGATGCGCAGAGTCTTG	85		
HSP90-beta	*HSP90B*	AF135117	CTCTGGGATGAGCTCCTCACA	CCTTTGACCTCTTTGAGAACAAGAA	98		
Elongation factor 1AA	*EF1AA*	AF321836	CCCCTCCAGGACGTTTACAAA	CACACGGCCCACAGGTACA	57		
Elongation factor 1AB	*EF1AB*	BG933853	TGCCCCTCCAGGATGTCTAC	CACGGCCCACAGGTACTG	59		
Metallothionein B	*MT-B*	CK990996	TGAATAAAGAAGCGCGATCAAA	CTGGTGCATGCGCAGTTG	111	1.8	1.5*
Fatty acid binding protein	*FABP2*	CB511332	TTACGGAAGGTGCTGGATTC	GGGCATCAATGTGATGAAGA	108	−2.2	−2.5*
Gamma crystallin M2	*CryG M2*	CB510188	GGAGAAGATGTCACGGTGGT	CCCCCTACAGAGGAGCCTAC	125	−1.8	−1.8*
Ependymin precursor	*EPN*	CA042089	TCCAGTTTAGCGTCCTGACA	GACAAGACCGGCTGGATACT	104	−1.7	−2.3*
Fructose-bisphosphate aldolase B	*Aldo b*	CA043730	CCTGTGTGGTCATCTCTCCAT	TGACAAGGGTGTCCTCTTCC	116	−1.7	−1.1
Sodium/potassium-transporting ATPase subunit alpha-1 precursor	*ATPA1C*	CA054630	AGGGAGACGTACTACTAGAAAGCAT	CAGAACTTAAAATTCCGAGCAGCAA	85	1.4	1.5*
Calpain small subunit 1	*CAPNS1*	CB505442	AGGCACCCAATGAAGTTGTC	CTCAGGGCTCATCTCCTCAC	144	1.4	1.5*
Glyceraldehyde-3-phosphate dehydrogenase	*GAPDH*	CA051897	GGTATCCCTTCATGGGTCCT	CGTGTCAGTGGTGGACCTAA	104	1.2	1.8*
Ubiquitin-conjugating enzyme E2 D4	*UbE2D4*	CB508919	CATTGCATACTTTTGAGTCCAATC	ACCCAGATGACCCCCTAGTC	101	−1.3	−1.4*
Cold-inducible RNA-binding protein	*Cirbp*	CA062835	TGAGCTTCGACACCACAGAG	ACGAGACCTTCCCGTTTCTT	104	1.3	1.1
Peroxiredoxin-6	*PRDX6*	CA062947	TCAGGGATGTTTGGAGGAAC	GGGGCGTAACTTTGATGAGA	126	−1.2	−1.1
SPARC precursor	*SPARC*	CA052160	CCAAGGCGATGTACTTGTCA	GGTTCCTGTCCCACACAGAG	117	1.3	1.3*
Lens fiber membrane intrinsic protein	*LIM2*	CA039129	CCAGGAAGTTCACCGTCACT	TAATCGCAGGCATTCTCTCC	147	−1.3	−1.3*
Heat shock cognate 70 kDa protein	*HSC71*	CA767816	ACCTCGTTGCACTTCTCCAG	GCAGCGTGACAAGGTCTCTT	138	1.2	1.2*
Ferritin. heavy subunit	*Ferritin H*	CA052852	GTGTGGGTCGTTGTGTTCAG	AGTGGGGTAGTGGTGTGGAG	110	1.2	−1
Phospholipid hydroperoxide glutathione peroxidase. mitochondrial precursor	*GPX4*	CB505439	GGCTGTTCCTTCATCCACTT	GCCAGGTACAGAGGTGGAAA	126	1.2	1.1

The first four rows in the table contain the selected reference genes that are not differentially expressed in lenses from the different dietary groups. Expression of the 16 transcripts in the subsequent rows was significantly different between the dietary groups when analyzed by microarray and SAM, but only 11 of the transcripts were significantly different when analyzed by qRT–PCR analysis and Mann–Whitney test (FCs are marked with an asterisk in the table).

Total lens RNA (500 ng) was reverse transcribed to cDNA using TaqMan Reverse Transcription Reagents (Applied Biosystems, Foster City, CA). Each RNA sample was reverse transcribed in triplicates. A standard curve composed of a six-point twofold serial dilution (1,000–31.25 ng) of a pool of all RNA samples was run in triplicates to calculate real-time PCR efficiencies for each gene. All cDNA samples were diluted 1:4 in Milli-Q water (Millipore, Billerica, MA). Real-time PCR was performed on 384 well plates in a reaction volume of 10 μl containing 1X Light Cycler 480 SYBR Green I Master (Roche Applied Science, Basel, Switzerland), gene specific primers (0.5 μM each), and 2 µl cDNA template. A melting curve analysis was applied to confirm the amplification of the expected gene-specific product.

The second derivative maximum method was applied to calculate crossing point (C_P_) values using the Lightcycler 480 Software (Roche Applied Science). C_P_ values were further converted into quantities using gene-specific efficiency values calculated from the standard curves according to the geNorm manual [29]. Dividing the mean of the triplicate quantities for each sample by a normalization factor led to mean normalized expression (MNE) values for the particular genes. The normalization factor was determined using the geNorm VBA applet for Microsoft Excel version 3.4 [29]. All four potential reference genes tested were highly stable with gene expression stability (M) values below 0.3, and hence all four were used to calculate the normalization factor.

### Statistical analysis

Differences in the lens NAH contents between LLL and MMM fish were tested by *t*-test, and differences in the cataract scores between LLL and MMM fish were tested by the Mann–Whitney test. Individual lens NAH concentrations were correlated to cataract scores by Spearman rank order test. The qRT–PCR data were analyzed by Mann–Whitney test, and correlation between fold change (FC) values obtained by microarray and qRT–PCR was tested by Spearman rank order test using GraphPad Prism version 5.01 for Windows (GraphPad Software, San Diego, CA). Correlation between individual cataract scores and gene expression values was tested by Spearman rank order test using the Statistica data analysis software system version 7.1. (StatSoft Inc., Tulsa, OK).

## Results

### Cataract scores and lens NAH concentrations

**Figure 1:**
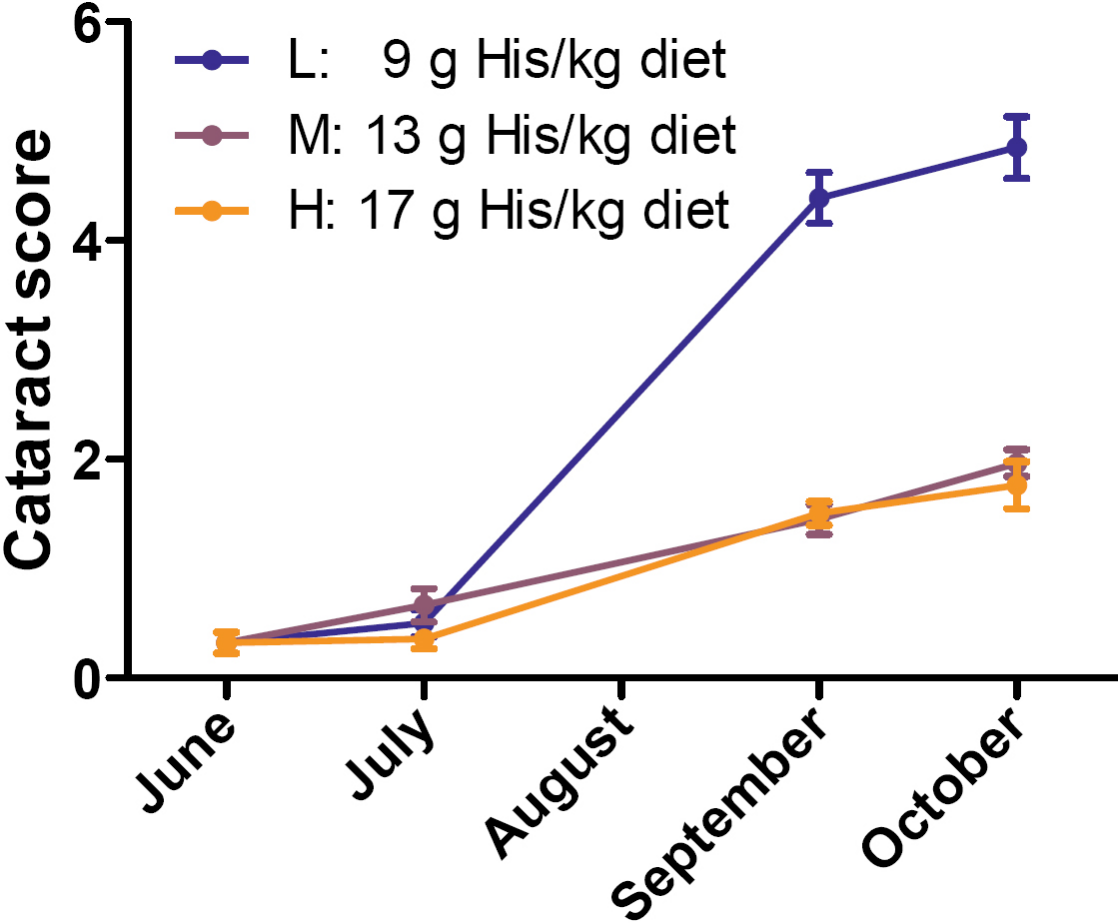
Cataract scores in selected dietary groups throughout the experimental period. The cataract score for each dietary group is given as the mean of the sums of the scores for both eyes, resulting in a possible maximum score of 8 (4 for each lens). Error bars show the standard error of the mean (SEM). The number of fish per group (n) varied from 31 to 113.

**Figure 2:**
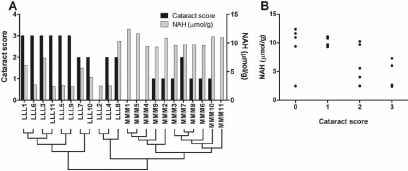
Individual cataract scores and N-acetyl histidine (NAH) concentrations in lenses of the fish used for microarray analysis. The right lens of the fish was used for microarray analysis, and thus the cataract scores (on a scale from 0 to 4) of the right lens are presented in the graphs. The NAH concentrations were determined in the left lens of the same fish. **A**: Cataract scores and NAH concentrations for the individual samples are shown in this graph. Under the sample names, the sample clustering (obtained by hierarchical clustering of genes and samples, see Figure 4) is shown to relate individual cataract scores and NAH concentrations to gene expression patterns (the closer the samples are in the cluster tree, the more similar is the lens transcriptome). **B**: Lens NAH concentrations were significantly negatively correlated to the cataract scores of the right lens (Spearman rank test; r=−0.63, p<0.002, n=22).

During the second and third experimental period, the fish developed cataracts with different severity depending on the dietary His regimes. Fish that were fed the low-His diet during the first and second period had a higher cataract frequency and severity than fish that were fed the medium- and high-His diet (Figure 1) [30]. The medium-His group was selected for microarray analysis to avoid possible negative effects of too high His concentrations in the high-His group. At the end of the trial, when samples for the microarray experiment were taken, there were significant differences in both cataract severity (Mann–Whitney test, p=0.001) and lens NAH concentration (*t*-test, p<0.001) between the low-His group and the medium-His group. The mean NAH concentration was 4.4±0.8 μmol/g (mean±SEM) in the low-His group and 10.4±0.3 μmol/g in the medium-His group. The individual single lens cataract scores and lens NAH concentrations are shown in Figure 2A. Lens NAH concentrations were significantly negatively correlated to the respective cataract scores (Spearman rank test; r=-0.63, p<0.002, n=22), which is shown in Figure 2B.

### Correspondence analysis plot

**Figure 3:**
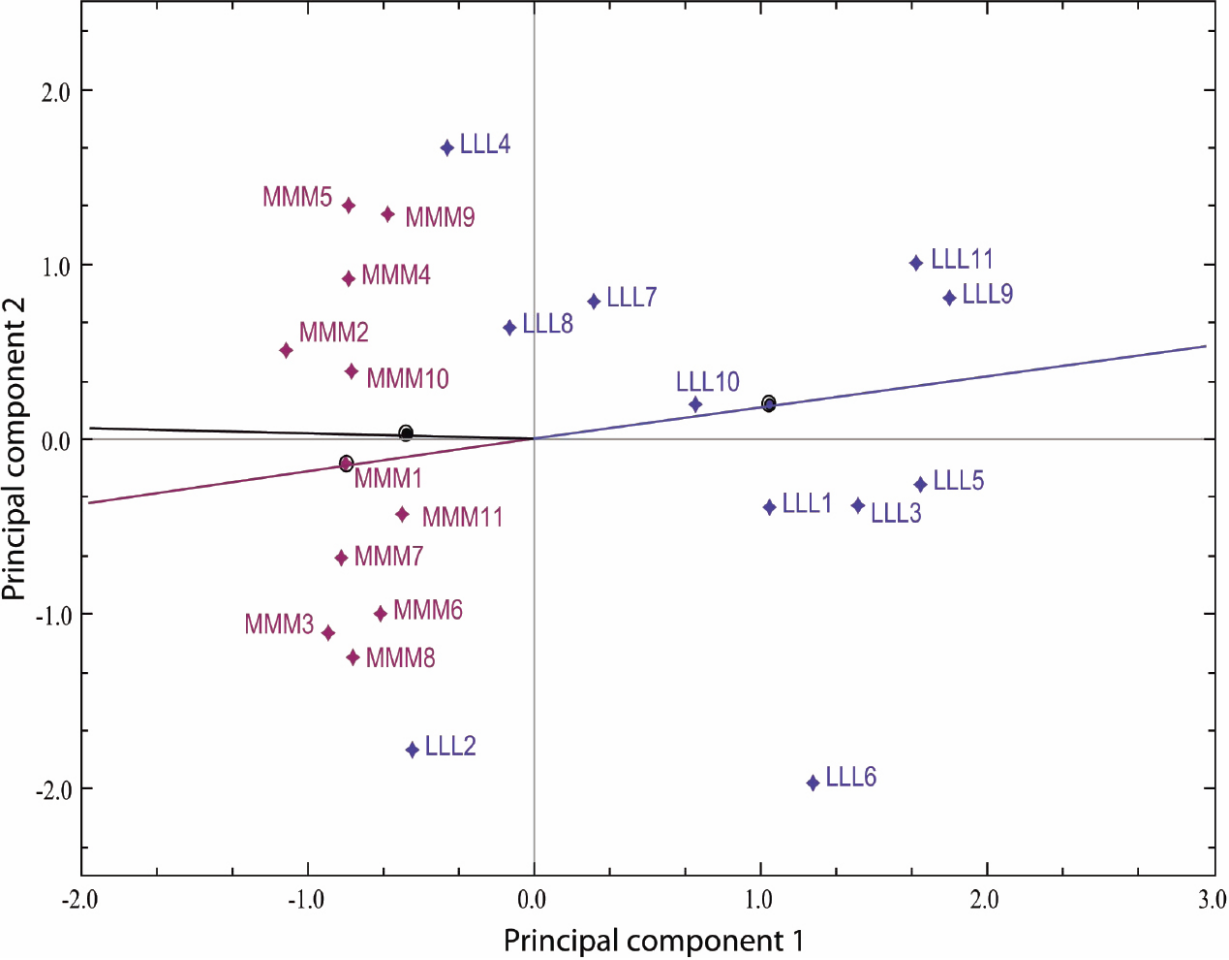
Correspondence analysis plot. The principal components 1 and 2, which explain the highest amounts of variance in the data set, are shown on the x- and y-axis of the plot, respectively. The samples are colored according to the dietary groups. The low-His samples (LLL) are blue, and the medium-His samples (MMM) are dark red. The dark red and blue lines are plotted from the point of origin through the respective group medians, which are marked by an equally colored dot. The total variance retained in the plot is 16.349%, the x-axis component variance is 10.623%, and the y-axis component variance is 5.726%.

Global differences in lens gene expression between the dietary groups were analyzed by microarray. After the pre-processing and filtering steps, the data set contained 4,242 transcripts. Correspondence analysis (CA) [21] was applied to look for associations between the samples and expression levels of the transcripts in the data set. Deviations from the null hypothesis (no association between samples and expression levels) add to the total χ^2^. This total χ^2 ^is decomposed in the CA plot shown in Figure 3 where the two largest dimensions, analogous to the principal components in factor analysis, are plotted on the x- and y-axis. The LLL and MMM samples were clearly separated along the first principal component (PC 1), which is the dimension explaining the largest amount of variance in the data set. The lines plotted from the point of origin through the group medians formed an angle of nearly 180°, indicating a clear separation of the dietary groups.

### Significance analysis of microarrays

Significance analysis of microarrays (SAM) [22] ranks the transcripts in a data set according to the regularized t-score that it calculates. It also provides a q value, which is a measure of the statistical significance of the differences in expression levels between the compared groups. The q value is a false discovery rate, which states the expected number of false positives on the list. In other words, SAM ranks the transcripts according to the significance of the difference in expression levels between the two dietary groups. On top of the SAM ranking list (Appendix 1) were 514 transcripts with a significant q value below 5%. Of these transcripts, 248 were up-regulated and 266 were down-regulated in the low-His group (LLL) compared to the medium-His group (MMM). Furthermore, 145 of these 514 transcripts had a highly significant q value of 0% (Table 2). Of these 145 transcripts, 59 transcripts were up-regulated and 86 were down-regulated in the low-His group compared to the medium-His group. The highest FC was 2.1 for the strongest up-regulated transcript and −2.5 for the strongest down-regulated transcript.

**Table 2:** SAM ranked gene list.

**Rank**	**Accession Number**	**Transcript name**	**d[i]**	**de[i]**	**Fold Change**	**q-value**	Regulation category*
1	CK990996	Metallothionein B	−7.557	−2.868	1.757	0	L
2	CA062118	PREDICTED: similar to POMT2 [Danio rerio]	5.977	2.89	−1.907	0	L
3	CA051877	UNKNOWN	5.144	2.66	−1.855	0	E
4	CB507722	Metallothionein B	−5.119	−2.649	1.58	0	E
5	CB493454	Lipocalin precursor	4.976	2.521	−1.666	0	E
6	CB498358	Fatty acid-binding protein. intestinal	4.875	2.439	−2.403	0	L
7	CA054659	Fatty acid-binding protein. intestinal	4.766	2.382	−2.539	0	L
8	CB492836	Lipocalin precursor	4.564	2.33	−1.639	0	L
9	CA064204	Protein S100-B	4.539	2.293	−1.579	0	L
10	CN442545	Cytochrome c oxidase subunit 3	−4.529	−2.528	1.504	0	L
11	CK990592	Metallothionein B	−4.493	−2.447	1.549	0	L
12	CB509992	Lipocalin precursor	4.439	2.256	−1.527	0	E
13	CB496407	Betaine aldehyde dehydrogenase	4.406	2.225	−1.824	0	L
14	CB515799	UNKNOWN	−4.403	−2.385	1.638	0	L
15	CA063208	UNKNOWN	4.387	2.194	−1.951	0	E
16	CA769320	Fatty acid-binding protein. intestinal	4.361	2.167	−2.247	0	L
17	CB498606	Fatty acid-binding protein. intestinal	4.328	2.142	−2.372	0	L
18	CB511332	Fatty acid-binding protein. intestinal	4.297	2.119	−2.156	0	L
19	CB492197	Metallothionein A	−4.276	−2.336	1.487	0	L
20	CB515213	B-cell linker protein	4.275	2.098	−1.604	0	L
21	CA046225	Metallothionein B	−4.218	−2.294	1.512	0	L
22	CA051958	Trafficking protein particle complex subunit 3	4.213	2.079	−1.6	0	L
23	CA051480	UNKNOWN	4.2	2.06	−2	0	L
24	CA044316	Excluded (Chimera)	4.196	2.044	−1.433	0	L
25	CB494699	Gamma crystallin M2	4.192	2.028	−1.544	0	L
26	CK990422	UNKNOWN	4.172	2.013	−1.669	0	L
27	CA059685	UNKNOWN	−4.163	−2.258	1.713	0	E
28	CB498630	Apolipoprotein Eb precursor	−4.161	−2.225	1.818	0	L
29	CA058895	Apolipoprotein Eb precursor	−4.07	−2.195	2.113	0	E
30	CA064247	CD9 antigen	4.068	1.998	−1.866	0	L
31	CA053993	UNKNOWN	4.062	1.985	−1.736	0	L
32	CB499689	Chromodomain-helicase-DNA-binding protein 2	4.059	1.971	−1.74	0	L
33	CA055129	UNKNOWN	4.045	1.958	−1.573	0	L
34	CA042615	SH3 domain-binding glutamic acid-rich-like protein	4.039	1.946	−1.528	0	L
35	CB510889	Putative polypeptide N-acetylgalactosaminyltransferase-like protein 4	4.022	1.935	−1.461	0	L
36	CA061651	PREDICTED: similar to calmodulin-dependent phosphodiesterase [Danio rerio]	3.961	1.924	−1.81	0	L
37	CA041385	Proactivator polypeptide precursor	−3.888	−2.169	1.396	0	E
38	CA042089	Ependymin precursor	3.868	1.913	−1.684	0	E
39	CB510842	Calcium-regulated heat stable protein 1	3.867	1.902	−1.952	0	L
40	CB492748	pigment epithelium-derived factor [Paralichthys olivaceus] >gi|71063313|gb|AAZ22324.1| pigment epithelium-derived factor [Paralichthys olivaceus]	3.866	1.892	−1.625	0	L
41	CA058611	Clusterin precursor	−3.863	−2.145	1.698	0	L
42	CB510615	UNKNOWN	3.852	1.883	−1.47	0	L
43	CA063671	UNKNOWN	−3.841	−2.123	1.751	0	L
44	CA042930	UNKNOWN	3.832	1.875	−1.612	0	L
45	CA062117	Protein FAM44B	3.795	1.865	−1.72	0	L
46	CN442558	Cytochrome c oxidase subunit 3	−3.745	−2.103	1.433	0	L
47	CA043114	Meprin A subunit alpha precursor	3.744	1.855	−1.738	0	L
48	CA043730	Fructose-bisphosphate aldolase B	3.74	1.847	−1.674	0	L
49	CB494396	Glycylpeptide N-tetradecanoyltransferase 1	3.717	1.839	−1.719	0	E
50	CB494172	Keratin. type II cytoskeletal 8	−3.716	−2.086	1.533	0	L
51	CA052024	UNKNOWN	3.709	1.83	−1.796	0	L
52	CB510653	Salvelinus alpinus metallothionein B gene. introns 1 and 2 and partial cds	−3.708	−2.068	1.376	0	L
53	CB506047	Thymosin beta-12	−3.674	−2.052	1.504	0	L
54	CA052938	UNKNOWN	3.663	1.822	−1.828	0	L
55	CA054630	Sodium/potassium-transporting ATPase subunit alpha-1 precursor	−3.66	−2.036	1.409	0	L
56	CB511371	Gamma crystallin M3	3.654	1.814	−1.584	0	L
57	CA063207	UNKNOWN	3.653	1.806	−1.776	0	L
58	CA055638	UNKNOWN	3.651	1.799	−1.497	0	L
59	CA044410	UNKNOWN	3.65	1.792	−1.561	0	L
60	CB498510	UNKNOWN	3.644	1.785	−1.652	0	L
61	CB510188	Gamma crystallin M2	3.643	1.778	−1.825	0	L
62	CB511962	Salmo salar TNF-alpha 2 gene. complete cds	3.613	1.77	−1.392	0	L
63	CN442525	ATP synthase a chain	−3.603	−2.02	1.298	0	L
64	CB512365	UNKNOWN	3.6	1.764	−1.451	0	E
65	CA047979	UNKNOWN	−3.599	−2.006	1.482	0	L
66	CB508872	GDP-L-fucose synthetase	−3.584	−1.992	1.388	0	L
67	CA056904	UNKNOWN	3.573	1.757	−1.75	0	L
68	CK990888	UNKNOWN	−3.567	−1.98	1.234	0	L
69	CA052962	Pentraxin fusion protein precursor	3.567	1.75	−1.639	0	L
70	CA062371	UNKNOWN	3.566	1.743	−1.622	0	L
71	CB517893	UNKNOWN	−3.559	−1.967	1.657	0	L
72	CB514528	Annexin A2-A	−3.543	−1.956	1.43	0	L
73	CB493750	Thymosin beta-12	−3.541	−1.944	1.53	0	L
74	CB505442	Calpain small subunit 1	−3.541	−1.932	1.395	0	L
75	CB502127	UNKNOWN	3.539	1.738	−1.836	0	L
76	CA039176	Gamma crystallin M2	3.538	1.732	−1.321	0	L
77	CB511903	Uncharacterized protein C1orf74 homolog	3.535	1.725	−1.682	0	L
78	CN442536	Cytochrome c oxidase subunit 3	−3.534	−1.921	1.3	0	L
79	CA063802	UNKNOWN	3.514	1.719	−1.455	0	L
80	CA047126	Glyceraldehyde-3-phosphate dehydrogenase	−3.499	−1.911	1.378	0	L
81	CA051479	UNKNOWN	3.47	1.714	−1.928	0	L
82	CA050751	Asparaginyl-tRNA synthetase. cytoplasmic	−3.47	−1.899	1.39	0	L
83	CA061252	Pleckstrin homology-like domain family A member 1	3.457	1.708	−1.62	0	L
84	CB502503	Cathepsin L precursor	3.453	1.702	−1.306	0	L
85	CB509531	Clusterin precursor	−3.447	−1.89	1.626	0	L
86	CA057824	Apolipoprotein Eb precursor	−3.436	−1.88	1.626	0	E
87	CA059732	Oncorhynchus mykiss SYPG1 (SYPG1). PHF1 (PHF1). and RGL2 (RGL2) genes. complete cds; DNaseII pseudogene. complete sequence; LGN-like. PBX2 (PBX2). NOTCH-like. TAP1 (TAP1). and BRD2 (BRD2) genes. complete cds; and MHCII-alpha and Raftlin-like pseudogenes. complete sequence	3.423	1.697	−1.716	0	L
88	CB513063	Perforin-1 precursor	3.417	1.692	−1.463	0	L
89	CA768741	UNKNOWN	−3.413	−1.871	1.357	0	L
90	CK990741	60S acidic ribosomal protein P1	3.398	1.687	−1.421	0	L
91	CB512134	Myosin light polypeptide 6	−3.388	−1.863	1.423	0	E
92	CB498494	Gamma crystallin M2	3.363	1.681	−1.345	0	L
93	CB502483	Fructose-bisphosphate aldolase B	3.358	1.676	−1.485	0	L
94	CA047466	UNKNOWN	3.344	1.671	−1.338	0	L
95	CB496999	UNKNOWN	−3.34	−1.854	1.273	0	L
96	CA060333	Hypoxanthine-guanine phosphoribosyltransferase	3.339	1.666	−1.267	0	L
97	CA062021	UNKNOWN	−3.323	−1.845	1.348	0	L
98	CA768027	Ubiquitin carboxyl-terminal hydrolase 32	3.321	1.661	−1.436	0	L
99	CA063261	Hexokinase-2	3.316	1.656	−1.633	0	L
100	CN442552	Cytochrome c oxidase subunit 3	−3.312	−1.837	1.334	0	L
101	CA042961	Eukaryotic peptide chain release factor subunit 1	−3.309	−1.829	1.345	0	L
102	CA057781	Nascent polypeptide-associated complex subunit alpha	3.308	1.651	−1.241	0	L
103	CA045638	Ubiquitin-conjugating enzyme E2 D4	3.304	1.646	−1.39	0	L
104	CA052800	THO complex subunit 1	3.285	1.641	−1.274	0	L
105	CB511609	Cathepsin L precursor	−3.279	−1.821	1.367	0	L
106	CB511219	Gamma crystallin M2	3.279	1.636	−1.504	0	E
107	CK990761	Cytochrome b	−3.275	−1.813	1.387	0	L
108	CA056981	UNKNOWN	3.274	1.631	−1.37	0	E
109	CA063526	Oncorhynchus mykiss G-protein (P-ras) mRNA. complete cds	3.263	1.627	−1.681	0	L
110	CN442497	Cytochrome c oxidase subunit 3	−3.259	−1.805	1.403	0	L
111	CA051104	Salmo salar TNF-alpha 2 gene. complete cds	3.247	1.622	−1.472	0	L
112	CA055845	UNKNOWN	3.244	1.618	−1.653	0	L
113	CA056167	Clusterin precursor	−3.209	−1.798	1.697	0	L
114	CA768207	Barrier-to-autointegration factor	3.206	1.613	−1.557	0	L
115	CA043681	UNKNOWN	−3.186	−1.791	1.25	0	L
116	CA047249	Excluded (Chimera)	−3.185	−1.784	1.63	0	L
117	CA052125	UNKNOWN	−3.185	−1.777	1.441	0	L
118	CB488101	Cold-inducible RNA-binding protein	−3.184	−1.77	1.35	0	L
119	CB515267	Integral membrane protein 2C	3.18	1.609	−1.436	0	L
120	CB499782	ADP-ribosylation factor 4	3.177	1.605	−1.585	0	L
121	CB492597	Ependymin precursor	3.17	1.6	−1.405	0	E
122	CK990775	UNKNOWN	−3.16	−1.763	1.291	0	L
123	CA051121	Homo sapiens calcium homeostasis endoplasmic reticulum protein (CHERP). mRNA	−3.157	−1.756	1.308	0	L
124	CB496460	hyperosmotic glycine rich protein [Salmo salar]	−3.147	−1.749	1.353	0	L
125	CB497026	Cathepsin L2 precursor	−3.146	−1.742	1.256	0	L
126	CA064587	UNKNOWN	3.146	1.596	−1.282	0	L
127	CA051534	UNKNOWN	−3.141	−1.736	1.391	0	L
128	CA052327	*Mus musculus* 10 days neonate skin cDNA. RIKEN full-length enriched library. clone:4732428C20 product:unclassifiable. full insert sequence	−3.138	−1.73	1.225	0	L
129	CB512146	B-cell linker protein	3.135	1.592	−1.468	0	L
130	CA041767	UNKNOWN	3.13	1.588	−1.399	0	L
131	CB497995	Eukaryotic translation initiation factor 4 gamma 1	3.127	1.584	−1.399	0	L
132	CA062835	Cold-inducible RNA-binding protein	−3.122	−1.725	1.297	0	L
133	CB514425	UNKNOWN	3.114	1.58	−1.383	0	L
134	CA037913	UNKNOWN	−3.096	−1.718	1.489	0	E
135	CB501344	Uncharacterized protein C8orf4 homolog	−3.095	−1.713	1.53	0	L
136	CA039269	UNKNOWN	3.094	1.576	−1.282	0	L
137	CK990381	Beta crystallin B3	3.092	1.572	−1.312	0	L
138	CK990533	Keratin. type II cytoskeletal 8	−3.077	−1.706	1.619	0	L
139	CA052374	UNKNOWN	3.071	1.568	−1.41	0	L
140	CA063499	UNKNOWN	3.055	1.564	−1.327	0	L
141	CA043660	Nuclear receptor 0B2	−3.044	−1.701	1.378	0	E
142	CA064148	UNKNOWN	−3.038	−1.696	1.271	0	L
143	CB494413	UNKNOWN	−3.009	−1.69	1.345	0	L
144	CB498981	RAB3A-interacting protein	3.003	1.561	−1.661	0	L
145	CA046217	UNKNOWN	−3.002	−1.685	1.357	0	L

The transcripts shown are those with a q-value of 0% with the most significantly differentially expressed transcripts on top of the list. The complete list is given in Appendix 1. Abbreviations used in the table header are: d[i], SAM score for a transcript i; de[i], expected SAM score for a transcript i. * The term "Regulation category" in the header of the last column refers to a categorization of the transcripts determined by appearance of the graphs resulting from correlation of expression levels to individual cataract scores (see Results section). The two categories are named E (“early” regulated transcripts), and L (“late” regulated transcripts).

### Hierarchical clustering

**Figure 4:**
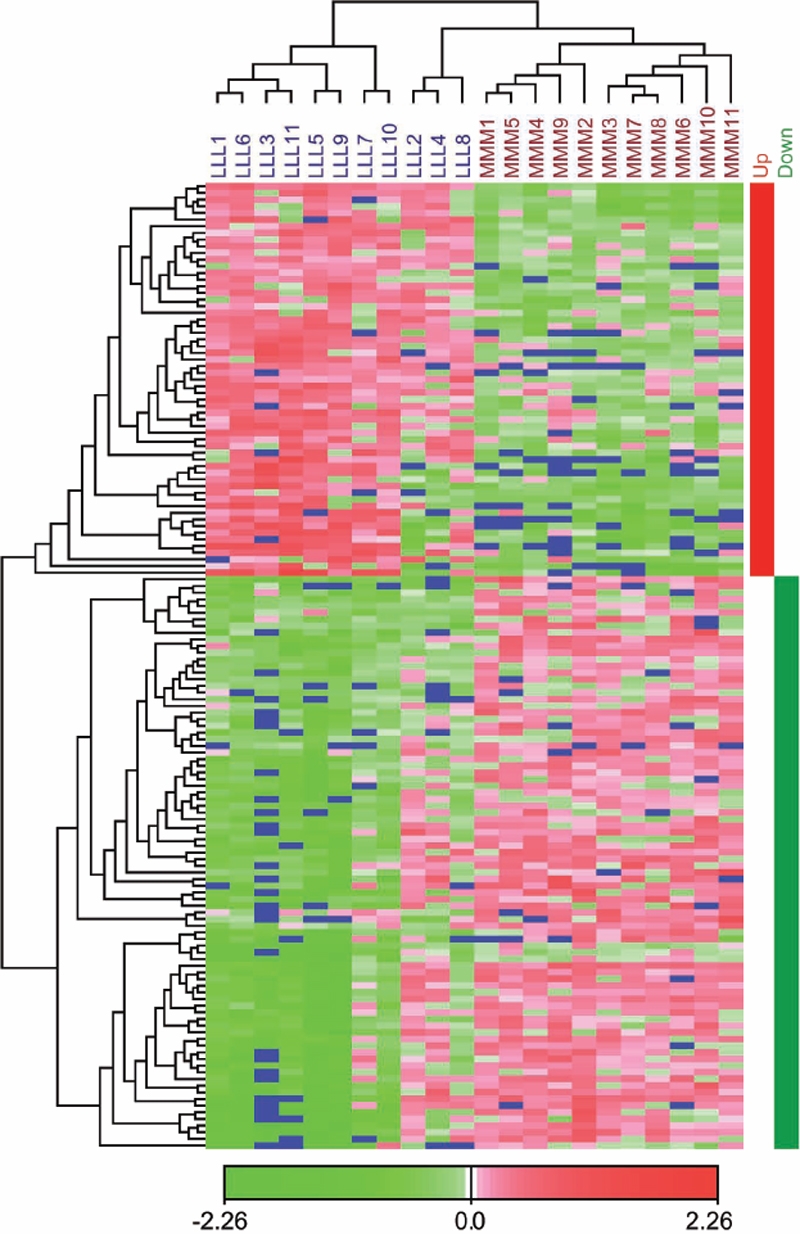
Hierarchical clustering of samples and transcripts. The samples are arranged in columns, and the transcripts are arranged in rows. Only the transcripts with a q-value of 0% in the SAM list were clustered. Negative log intensity ratios are shown in green and positive log intensity ratios are shown in red in the heat map as indicated by the color bar. The blue color represents missing values. The transcripts divide into two distinct clusters. The first cluster contains the transcripts that are up-regulated in the low-His group compared to the medium-His group and is marked by a red bar at the right side of the heat map. The second cluster contains the down-regulated transcripts and is marked by a green bar at the right side of the heat map. The samples divide into three main clusters, reflecting the His feeding regimes. Low-His samples are clearly separated from medium-His samples.

Hierarchical clustering of samples and transcripts was performed with the most significantly differentially expressed transcripts in the SAM top list including transcripts with q=0% (Figure 4). The transcripts (on the left side of the heat map) are clustered into two main groups, transcripts up-regulated in the low-His group and transcripts down-regulated in the low-His group. The samples (on top of the heat map) are arranged into three main clusters, representing the two dietary groups. The samples LLL2, LLL4, and LLL8 formed a main cluster together with the MMM samples with many transcripts displaying similar expression levels in this main cluster, which is shown by similar colors. In Figure 2A, the sample clustering is shown in relation to individual cataract scores and lens NAH concentrations to visualize the interactions between lens His status, cataract scores, and gene expression patterns in individual fish of the two dietary groups. There are three main clusters, cluster 1, cluster 2, and cluster 3. While cluster 1 including samples LLL1, LLL6, LLL3, LLL11, LLL5, LLL9, LLL7, and LLL10 is relatively uniform with high cataract scores and low NAH concentrations, cluster 2 with samples LLL2, LLL4, and LLL8 shows both high and low cataract scores and high and low NAH concentrations. In cluster 3 containing all MMM samples, NAH concentrations are equally high, and cataract scores are relatively low.

### Functional enrichment analysis using Blast2GO

Functional enrichment analysis was performed using the Blast2GO platform with the aim to see if groups of transcripts belonging to the same functional classes were enriched among the most significantly differentially expressed transcripts. The top of the SAM list (including transcripts with q<5%) was compared to the complete SAM list. The complete analysis results can be found in (Appendix 2). Among others, the functional categories described by the following Gene Ontology (GO) terms were enriched with a false discovery rate (FDR) of less than or equal to 5% (with the respective transcript names): “Cysteine-type endopeptidase activity” (Calpain small subunit 1, Cathepsin L precursor, Ubiquitin carboxyl-terminal hydrolase 32, Cathepsin L2 precursor, Calpain-2 catalytic subunit precursor, Cathepsin B precursor, Calpain-2 catalytic subunit), “Glycolysis” (Fructose-bisphosphate aldolase B, Glyceraldehyde-3-phosphate dehydrogenase, Hexokinase-2, Triose phosphate isomerase), and “Lipid metabolic process” (Lipocalin precursor, Clusterin precursor, Sodium/potassium-transporting ATPase subunit alpha-1 precursor, Peroxiredoxin-6, Proactivator polypeptide precursor, Fatty acid binding protein 3 (FABP3), Phospholipid hydroperoxide glutathione peroxidase, mitochondrial precursor, Triose phosphate isomerase, Acyl-CoA-binding protein, Prostaglandin E synthase 3, Diacylglycerol O-acyltransferase 2).

### Correlation of gene expression to cataract score and lens NAH concentrations

**Figure 5:**
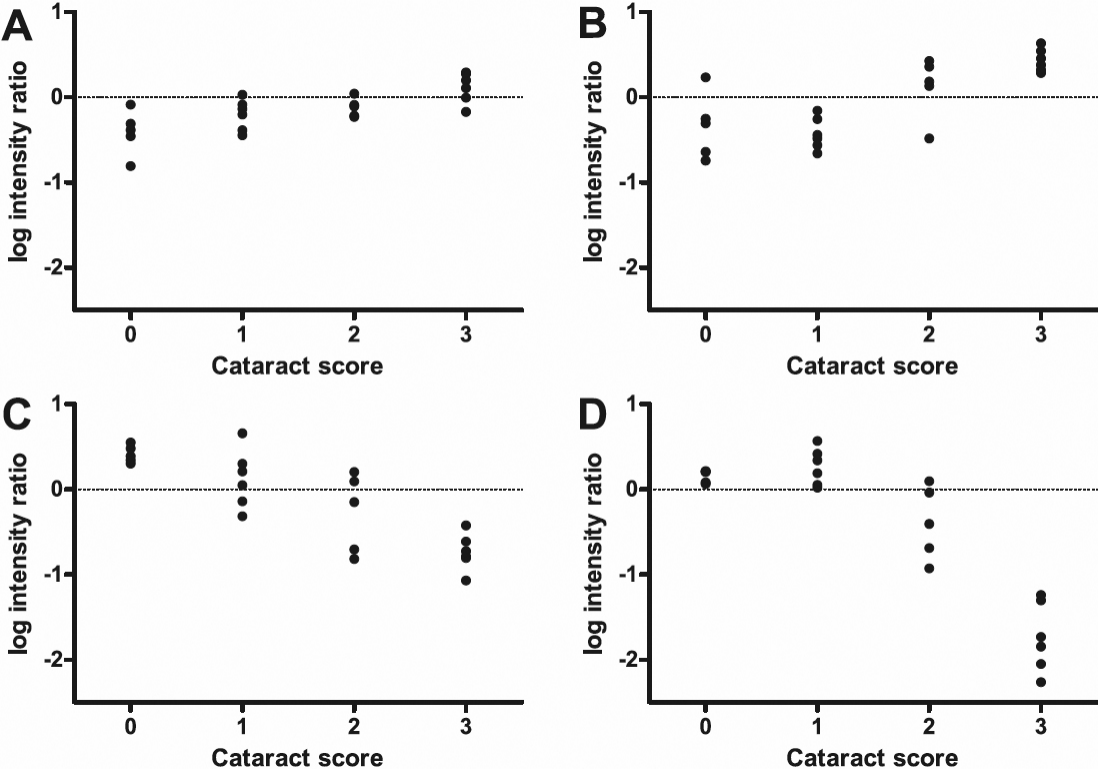
Examples of transcripts with different expression patterns related to cataract score. For four selected significantly differentially expressed transcripts, the log intensity ratios are plotted against the cataract score of the respective sample, not taking into account which dietary group the samples belong to. For a certain transcript, if the difference between the mean log intensity ratios of the lenses with a score of 0 and the lenses with a score of 1 was 0.2 or greater, this transcript was classified as “early” regulated. If this difference was less than 0.2, the transcript was classified as “late” regulated. **A**: SPARC precursor (SPARC; CA052160) was chosen as an example for “early” up-regulated transcripts. **B**: Metallothionein B (MT-B; CK990996) was chosen as an example of “late” up-regulated transcripts. **C**: Ependymin (EPN; CA042089) was chosen as an example of “early” down-regulated transcripts. **D**: Fatty acid binding protein 2 (FABP2; CA054659) was chosen as an example of “late” down-regulated transcripts.

In the microarray experiment, we statistically compared samples from different dietary groups. To further elaborate the results of the microarray experiment, we correlated individual gene expression data of the 145 highly significantly differentially expressed transcripts (q=0% in the SAM top list) to the cataract score and the NAH concentration of the respective lens. The expression of most of the transcripts (99%) was significantly correlated to the cataract score (Spearman rank test; p<0.05, n=22). Similarly, expression of 94% of the transcripts was significantly correlated to the lens NAH concentration (Spearman rank test; p<0.05, n=22). According to their expression pattern relative to the cataract score, the transcripts could roughly be divided into two regulation categories, “early” regulated and “late” regulated transcripts. To illustrate the observed patterns, Figure 5 shows graphs for SPARC precursor (*SPARC*), metallothionein B (*MT-B*), ependymin (*EPN*), and fatty acid binding protein 2 (*FABP2*).

For the “early” regulated transcripts, the expression levels changed continuously from lenses with cataract score 0 to the highest observed cataract score, which was 3. To distinguish between “early” and “late” regulation for a transcript, we used the difference between the mean log intensity ratios of the lenses that scored 0 and the lenses that scored 1. For “early” regulated transcripts, we defined this difference to be 0.2 or greater. “Early” regulated transcripts had either consistently increasing (Figure 5A) or decreasing (Figure 5C) expression levels, or had a maximum in lenses with cataract score 1 and decreasing expression levels at the higher cataract scores (data not shown). In contrast, for the “late” regulated transcripts, there were no apparent differences in expression levels between lenses with a score of 0 and lenses with a score of 1 (the differences in log intensity ratios between the mean of the lenses with score 0 and the mean of the lenses with score 1 were less than 0.2). With more severe cataracts, i.e., higher cataract scores, the expression levels increased (Figure 5B) or decreased (Figure 5D).  Appendix 1 and Table 2 summarize which type of regulation category the transcripts with q=0% in the SAM top list could be assigned to. The majority of the transcripts (88%) were found to be “late” regulated.

### Validation

**Figure 6:**
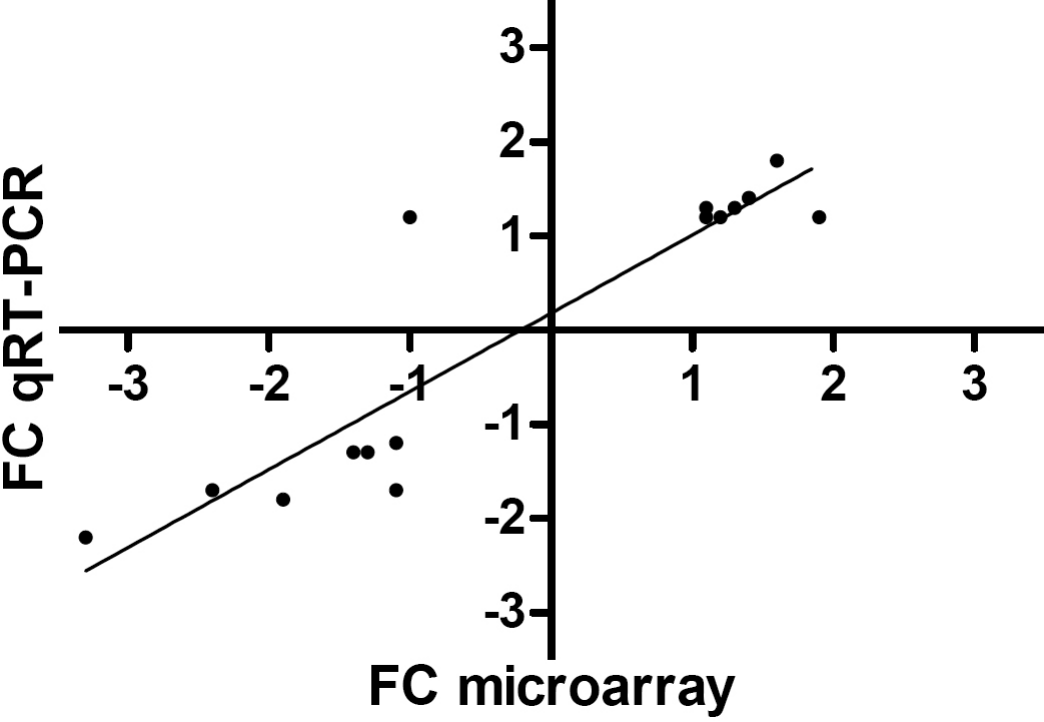
Correlation between fold change values obtained by microarray analysis and qRT–PCR for 16 selected transcripts. Fold change (FC) values obtained by microarray analysis were significantly correlated to those obtained by qRT–PCR (Spearman rank test; r=0.89, p<0.0001, n=16).

From the transcripts that were significantly up-regulated or down-regulated (q<5%) in the low-His group when compared to the medium-His group in the microarray experiment, we selected sixteen EST clones for qRT–PCR validation. Eleven of these sixteen transcripts were significantly differentially expressed between the two dietary groups when tested by qRT–PCR and Mann–Whitney test, thereby confirming the microarray results. The FC values obtained by microarray and qRT–PCR analysis are listed in Table 1. The FC values of the qRT–PCR results were calculated based on the median of the MNE values of the samples in both dietary groups. There was a significant correlation between the FC values obtained by microarray and qRT–PCR analysis (Spearman rank test; r=0.89, p<0.0001, n=16; Figure 6).

## Discussion

### The occurrence of cataracts in Atlantic salmon is related to dietary histidine

The present feeding experiment showed that adult Atlantic salmon in sea water that were fed a low-His diet during the first experimental period from June to July and/or the second period from July to September developed severe cataracts, appearing mainly after the second period (Figure 1). However, the levels of dietary His did not affect the growth of the fish in the different feeding groups during the trial [30]. Since there were no other known variables in this feeding experiment, the differences in cataract development and gene expression observed between the dietary groups are assumed to be solely due to dietary His feeding regimes. This assumption was supported by the concentration differences of the His derivative NAH in the lenses (Figure 2A), the concentration of imidazoles in muscle tissue [30], and the strong negative correlation between individual lens NAH concentrations and cataract scores (Figure 2B). A similar His-related cataract development was reported in younger Atlantic salmon smolt after sea transfer [9]. The current His minimum requirement for Atlantic salmon is estimated to be 7 g/kg diet [5]. All experimental diets contained more than 7 g/kg diet. Even so, the incidences of cataract observed during this trial strongly indicate that the current theoretical His minimum requirement level is not sufficient to prevent development of cataracts in adult Atlantic salmon in their second year in sea water.

### Methodological considerations on the microarray experiment

The present microarray study was undertaken to explore molecular events connected to the observed dietary His-related cataract development in Atlantic salmon. Interpretation of the microarray data by correspondence analysis, SAM, and hierarchical clustering revealed clear differences in the lens transcriptome between the compared dietary groups. Both by CA of the whole data set (Figure 3) and hierarchical clustering of the significantly differentially expressed transcripts (Figure 4), the three samples LLL2, LLL4, and LLL8 were situated close to the MMM samples, showing that this relation is not only restricted to the highly significantly differentially expressed transcripts. In these three samples, the expression patterns of the significantly differentially expressed transcripts were similar (Figure 4), but the lens NAH concentrations and cataract scores were different (Figure 2A). This might indicate that gene expression is also influenced by individual predisposition.

Our findings clearly confirm that changed gene expression is involved in the process of His-related cataract development. Although the differences in the expression levels, given as fold change (FC) values, were generally low, a considerable number of transcripts were found to be significantly differentially expressed. The main cataract outbreak was registered in the period from July to September, while lenses for the microarray experiment were sampled in October. Some of the observed differences in gene expression levels might thus reflect secondary and compensatory reactions to pathophysiologic changes in the lenses over a longer period of time rather than processes directly involved in cataract development.

In contrast to the human genome and to model species like the mouse and rat, the annotation of the salmon genome is rather poor. The salmonid microarray used in the study is based on EST clones, and the annotation is mainly based on sequence similarities to other species [17]. Only few transcripts on the array are characterized in salmonids. The SAM list contained about 25% uncharacterized EST clones (named UNKNOWN in Appendix 1 and Table 2) while 75% had been identified and were annotated with a transcript name. Using Blast2GO, approximately 67% of these identified transcripts (about 50% of all transcripts in the SAM list) were functionally annotated with at least one GO term. This left about 25% of the transcripts in the SAM list identified but without functional annotation. These 25% were not included in the functional enrichment analysis. An example for this is the intestinal type fatty acid binding protein (*FABP2*), which was not included in the functional category “Lipid metabolic process”, although it is known to be involved in lipid metabolic processes. Thus, the functional enrichment analysis results do not give a complete view of the functional categories enriched in the data set.

### Selected differentially expressed transcripts and their possible role in His-related cataract

The first transcript in the SAM list (Table 2) is an EST clone coding for metallothionein B (*MT-B*). Metallothionein A (*MT-A*) is number 19 in the list. Both isoforms are up-regulated in the low-His group. Metallothioneins are multifunctional stress proteins induced by a variety of stresses. They can take part in the detoxification of heavy metals, the regulation of zinc levels in the cell, and the protection against oxidative stress [31]. While heavy metals such as cadmium [32] and lead [33] have been linked to cataract development, oxidative stress is generally one of the major factors associated with cataracts [34,35]. Direct evidence of the involvement of metallothioneins in cataract-related processes has been given by the study of Kantorow et al. [36], who detected increased levels of metallothionein IIA transcripts in human age-related cataractous lenses relative to clear lenses by RT–PCR differential display. Hawse et al. [37] showed later that metallothionein IIA defended lens epithelial cells against oxidative stress induced by cadmium and tertiary butyl hydroperoxide.

We hypothesized that oxidative stress related to cataract triggered the increased expression of metallothioneins in the low-His group and assumed that other antioxidant genes present in the data set might be regulated similarly to *MT-B* and *MT-A*. Other transcripts with an antioxidant function that are present in the SAM list with q<5% were peroxiredoxin-6 (*PRDX6*), phospholipid hydroperoxide glutathione peroxidase (*GPX4*), selenoprotein Pb precursor, and thioredoxin (*TRX*; Appendix 1 and Table 2). Only *GPX4* was up-regulated. Although significant with fold changes around 1.2, none of the above mentioned antioxidant genes were as clearly regulated as the metallothionein transcripts. In contrast to our first hypothesis, down-regulation of antioxidant genes could lead to decreased antioxidant capacity and increased oxidative stress, which might in turn lead to cataract development. This effect might have been observed in a study on the impact of elevated water oxygen levels on cataract formation in smolting salmon [38]. The authors found trends of down-regulation of the antioxidant genes Cu/Zn superoxide dismutase (*Cu/Zn SOD*) and glutathione S-transferase (*GST*) in lenses of the treatment groups that developed more severe cataracts. Despite the suggested antioxidative properties of imidazoles, we cannot conclude which role oxidative stress might play in the present His-related cataracts observed in Atlantic salmon. More confirmative work has to be done to address this question. It also has to be considered that the expression of the stress-responsive antioxidant genes is often rapidly regulated upon the inducing stress. The fact that the development of cataracts in our study probably was more like a chronic stress to the lens further complicates the interpretation of the results.

One of the functional categories of transcripts revealed by functional enrichment analysis was related to lipid metabolism. Among the strongest down-regulated transcripts in the low-His group were lipocalin precursor (presumably coding for a lipocalin-type prostaglandin-D synthase, *Ptgds*) and the intestinal type fatty acid binding protein (*FABP2*). *Ptgds* is one of the most abundantly expressed transcripts in human [39] and zebrafish (*Danio rerio*) [40] lenses. *Ptgds* has two functions, the synthesis of the prostaglandin PGD_2_ in several tissues and binding to a variety of lipophilic ligands like biliverdin, bilirubin, retinaldehyde, and retinoic acid [41]. PGD_2_ is the major prostaglandin in the central nervous system and is involved in numerous physiologic functions. In the eye, PGD_2_ lowers the intraocular pressure and triggers inflammatory effects on the conjunctiva [42]. Cataract formation in lens epithelial cells is preceded by programmed cell death (apoptosis) [43]. Ptgds has been shown to protect neurons and oligodendrocytes against apoptosis in a mouse model of a demyelinating disease [44], but also a pro-apoptotic function has been reported [45]. The exact role of Ptgds in the cataractous salmon lens remains to be identified, but it might be involved in lens compensation mechanisms and repair.

FABP2 belongs to the fatty acid binding proteins. The members of this protein family are generally thought to facilitate lipid transport in the cell but may also be involved in lipid signaling pathways [46]. Fatty acid binding protein subtypes are expressed in numerous tissues. The expression of more than one subtype in a cell type indicates specific functions of the subtypes. Our data indicates the expression of *FABP2*, *FABP3*, *FABP4*, and *FABP7* in the salmon lens (Appendix 1). In bovine, human, and rat lenses, the expression of the epidermal type fatty acid binding protein (*FABP5*) has been demonstrated [47,48]. It has recently been shown that FABP2 stimulates mitochondrial β-oxidation and affects the cellular cholesterol transport in human intestine epithelial cells [49]. Given a similar function in the salmon lens, the decreased expression of *FABP2* would enhance cholesterol absorption and decrease fatty acid oxidation, leading to decreased energy production in the lenses of fish in the low-His group.

In contrast to *Ptgds* and *FABP2*, apolipoprotein Eb (*Apo Eb*) and clusterin precursor were up-regulated in the low-His group. Apo Eb serves as an extracellular transport protein for cholesterol and other lipids via binding to low density lipoprotein (LDL) receptors on the target cell surface, but also functions in repair response to tissue injury, immunoregulation, and modulation of cell growth and differentiation have been reported [50]. Expression of *Apo Eb* is activated by peroxisome proliferator-activated receptor γ (PPARγ) [51]. Clusterin is associated with high density lipoprotein (HDL) in the plasma and is also called apolipoprotein J [52,53]. Clusterin is up-regulated in developmental remodeling, apoptotic states in neurodegeneration, response to injuries, and other stresses and interacts with a variety of molecules [54]. Its expression is regulated by the heat shock transcription factor, HSF-1, and clusterin was proposed as an extracellular chaperone [55]. A truncated form acts as a death signal in the nucleus [56] while the normal secreted form promotes cell survival [57,58]. In the cataractous salmon lens, Apo Eb and clusterin might possibly play a role in tissue repair, similar to what is observed in nerve tissue.

However, a pure lipid transporting role of this group of transcripts, which maintain the cellular lipid homeostasis, is supported by the fact that the water temperature at the research station rose from 10 °C to 20 °C from June to July. As an adaptation to the environmental temperature changes, the membrane lipid composition in poikilotherms is changed to maintain fluidity and thus proper function [59]. A rapid and strict regulation of the membrane lipid composition in the salmon lens is assumed to be essential to keep the crystalline lens clear. A temperature-induced change in expression levels, however, would be expected to have declined after three months. Nevertheless, there is some evidence in the literature for the importance of lipids in relation to cataracts. Atlantic salmon that were fed diets based on plant lipid sources in a full life cycle feeding experiment seemed to be more prone to cataract development than fish that were fed diets based on conventional lipids of marine origin [60]. Similarly, age-related cataracts in humans have been related to dietary fat intake. Elevated intakes of 18:2n-6 (linoleic acid) and 18:3n-3 (α-linolenic acid) may increase the risk for cataract [61], while higher intakes of n-3 polyunsaturated fatty acids (PUFA) from fatty fish consumption may contribute to cataract prevention [62]. The study of lipid-related processes in the Atlantic salmon lens will be important to resolve processes leading to cataract development, especially seen in the light of the increasing importance of alternative sustainable lipid sources like plant oils in fish feed.

Glucose is the main energy source for the lens [1]. Several transcripts involved in carbohydrate metabolism were differentially regulated in the low-His group: fructose-bisphosphate aldolase B (down), glyceraldehyde-3-phosphate dehydrogenase (up), hexokinase-2 (down), and triose phosphate isomerase (up). The encoded proteins are all part of the glycolytic pathway, and three of them can also catalyze the reverse reaction in gluconeogenesis. The fourth, hexokinase-2, catalyzes the first step in the glycolytic pathway, and its down-regulation thus indicates a decrease in the energy-producing glycolytic activity in the low-His group. A central enzyme in the non-oxidative pentose phosphate shunt, transaldolase, was also down-regulated in the low-His group. The pentose phosphate shunt is the main source for reduced coenzyme, nicotinamide adenine dinucleotide phosphate (NADPH), which is needed for lipid biosynthesis and to regenerate oxidized glutathione, the major antioxidant in the lens [63,64]. The lack of reduced glutathione might critically impair the redox state of the lens cells and thus promote oxidative damage leading to cataract.

One transcript up-regulated in the low-His group was an EST clone encoding the α-1 subunit of Na^+^/K^+ ^ATPase. Na^+^/K^+ ^ATPase plays an important role in the regulation of the Na^+^ and K^+^ ion balance and thus the osmotic balance in cells, which is especially important to maintain transparency in the lens. Different Na^+^/K^+^ ATPase subunit isoforms are expressed in lenses of different species, and the isoform expression pattern is also specific for cell type and localization in the lens [65]. There are at least five isoforms of the α subunit in Atlantic salmon [66]. In several studies, Na^+^/K^+^ ATPase has been found to be involved in cataract-related processes. Its activity was impaired by H_2_O_2_-induced oxidative stress in cultured bovine lenses [67] and by the lipid peroxidation product, 4-hydroxynonenal [68]. In cataractous human lenses, the Na^+^/K^+^ ATPase activity was found to be decreased [69], and inhibition of the Na^+^/K^+^ ATPase activity has been shown to increase opacity in cultured rat lenses [70]. Disturbance of the ion balance by the ionophore, amphotericin B, led to increased Na^+^/K^+^ ATPase α-2 expression in porcine lens epithelium [71]. The upregulation of Na^+^/K^+^ ATPase α-1 seen in our study might be a sign of disturbed lens ion balance in the low-His group. NAH has been suggested as a major osmolyte in the fish lens [7,10], and the low concentrations of NAH in the low-His group (Figure 2A) suggest a lower preparedness to osmotic challenges and impacts on other actors in osmoregulation. The activity of Na^+^/K^+^ ATPase is also very energy-demanding, and the increased expression might be an attempt to compensate for decreased enzymatic activity caused by the lack of energy, resulting from the indicated decrease in glycolytic activity in the low-His group.

Several proteases were up-regulated in the low-His group, the regulatory and catalytic subunits of calpain and cathepsin L and B. Calpain is a calcium-dependent neutral protease that plays a role in the process of apoptosis [72]. Apoptosis has been related to cataract [43], and calpain has been found to be activated in various types of cataracts in rodents [73-75]. Possible calpain substrates in the lens are β-crystallins [76] and aquaporin 0, the main water channel in the lens [77]. Cathepsins are lysosomal cysteine proteases that participate in the degradation of structural proteins in the post-mortem muscle of salmon [78]. Cathepsins also seem to be involved in cataract-related processes since cathepsin A activity in the aqueous humor of cataract patients was found to be increased when compared to the aqueous humor of patients with other ocular diseases [79]. These proteases most probably play roles in secondary repair processes in the cataractous salmon lenses.

### Potential early cataractogenesis markers

In addition to conventional microarray data analysis, we explored our data further by correlating individual gene expression values directly to the respective cataract scores and lens NAH concentrations without considering the dietary background. The results of this approach strengthen our findings and confirm the role of lens NAH as a marker for dietary His levels and the impact of dietary His regimes on lens gene expression. According to their expression pattern relative to cataract score, the transcripts could roughly be divided into two regulation categories, “early” regulated and “late” regulated transcripts (Figure 5). “Early” regulated transcripts are probably more directly involved in or affected by cataract development and might be used as biological markers for early cataract detection in future experiments. “Late” regulated transcripts might be induced or repressed by secondary changes and compensatory mechanisms in the cataractous lens. One of the “early” up-regulated transcripts is SPARC, which is also among the most abundant transcripts in the zebrafish lens [40]. SPARC is an extracellular matrix-associated glycoprotein with multiple functions in tissue development and remodeling, cell turnover, and tissue repair [80,81]. Kantorow and coworkers [82] detected increased levels of SPARC transcripts in cataractous lenses when compared to normal lenses, and the same was shown on the protein level [83]. SPARC was also increased in cataractous lenses when compared to normal lenses as revealed by a microarray study [84]. Deletion of SPARC in mice leads to cataract development [85,86]. Emerson and coworkers [87] proposed a chaperone-like activity for SPARC.

One of the “early” down-regulated transcripts is ependymin. Ependymin is a glycoprotein and a major component of the brain extracellular fluid of goldfish (*Carassius aureatus*) and is involved in neuroplasticity, memory and learning, and tissue regeneration [88]. Its expression is induced by cold in zebrafish and carp (*Cyprinus carpio*) brain [89]. A trypsin-derived peptide fragment of ependymin activates the transcription factor, AP-1, in mouse neuroblastoma cells [90] and increases the expression of the antioxidant enzymes superoxide dismutase (*SOD*), catalase (*CAT*), and glutathione peroxidase (*GPX*) in rat primary cortical cultures [91]. As mentioned earlier, trends of down-regulation of the antioxidant genes, *Cu/Zn SOD *and *GST*, were observed in smolting salmon that were developing cataracts after exposure to elevated water oxygen levels [38]. Further dedicated experiments must be undertaken to strengthen and verify the indications we obtained by relating gene expression levels directly to cataract scores, and to establish the proposed transcripts (or corresponding functional analyses) as markers for early cataractogenesis.

Dietary histidine regimes affected cataract formation in adult Atlantic salmon and lens gene expression. Among the differentially expressed transcripts found in this study were metallothionein A and B as well as transcripts involved in lipid metabolism, carbohydrate metabolism, regulation of ion homeostasis, and protein degradation. In addition to providing new directions for cataract research in Atlantic salmon, the results of this genome-wide transcription analysis allowed us to suggest selected transcripts as possible biological markers for early cataract diagnosis in Atlantic salmon and with a potential for use in mammalian experiments.

## Appendix 1. SAM complete ranked gene list.

To access the data, click or select the words “Appendix 1.” This will initiate the download of a compressed (pdf) archive that contains the file. The most significantly differentially expressed transcripts are on top of the list. Transcripts with a q-value under 5% are regarded significantly differentially expressed between the two dietary groups. Abbreviations used in the table header are: d[i], SAM score for a transcript i; de[i], expected SAM score for a transcript i. * The term "Regulation category" in the header of the last column refers to a categorization of the transcripts determined by appearance of the graphs resulting from correlation of expression levels to individual cataract scores (see Results section). The two categories are named E (“early” regulated transcripts), and L (“late” regulated transcripts).

## Appendix 2. Functional enrichment analysis.

To access the data, click or select the words “Appendix 2.” This will initiate the download of a Microsoft Excel worksheet (xls) file. Functional annotations for the transcripts in the data set were assigned using the Blast2GO software. We compared transcripts with  q-values less than 5% in the SAM top list to the complete SAM list to find functional categories overrepresented among the significantly differentially expressed transcripts. Listed are functional categories enriched with a false discovery rate (FDR) of 0.5 or below. In the table headings, “Test” stands for the tested SAM top list with q-values of less than 5%, and “Ref” stands for the remaining transcripts in the SAM list. FWER: family wise error rate.
